# Role of the eNOS Uncoupling and the Nitric Oxide Metabolic Pathway in the Pathogenesis of Autoimmune Rheumatic Diseases

**DOI:** 10.1155/2020/1417981

**Published:** 2020-04-13

**Authors:** Anna Łuczak, Marta Madej, Agata Kasprzyk, Adrian Doroszko

**Affiliations:** ^1^Department of Rheumatology, Wroclaw Medical University, Poland; ^2^Department of Internal Medicine, Hypertension and Clinical Oncology, Wroclaw Medical University, Poland

## Abstract

Atherosclerosis and its clinical complications constitute the major healthcare problems of the world population. Due to the central role of endothelium throughout the atherosclerotic disease process, endothelial dysfunction is regarded as a common mechanism for various cardiovascular (CV) disorders. It is well established that patients with rheumatic autoimmune diseases are characterized by significantly increased prevalence of cardiovascular morbidity and mortality compared with the general population. The current European guidelines on cardiovascular disease (CVD) prevention in clinical practice recommend to use a 1,5-factor multiplier for CV risk in rheumatoid arthritis as well as in other autoimmune inflammatory diseases. However, mechanisms of accelerated atherosclerosis in these diseases, especially in the absence of traditional risk factors, still remain unclear. Oxidative stress plays the major role in the endothelial dysfunction and recently is strongly attributed to endothelial NO synthase dysfunction (eNOS uncoupling). Converted to a superoxide-producing enzyme, uncoupled eNOS not only leads to reduction of the nitric oxide (NO) generation but also potentiates the preexisting oxidative stress, which contributes significantly to atherogenesis. However, to date, there are no systemic analyses on the role of eNOS uncoupling in the excess CV mortality linked with autoimmune rheumatic diseases. The current review paper addresses this issue.

## 1. Introduction

Atherosclerosis and its clinical complications constitute the major healthcare problems of the world population [1–3]. Over the last decades, it has become clear that the vascular endothelium plays the central role throughout the atherosclerotic disease process, and all alterations initiating the onset and promoting the progression of the disease depend on the dynamic changes in endothelial cell phenotype. Endothelial dysfunction (ED), the early feature of atherosclerosis, precedes the development of morphologic changes and is the earliest detectable impairment of vascular function [4, 5]. It is a consequence of chronic exposure to cardiovascular (CV) risk factors, and its progression is related to the intensity and duration of these factors [6, 7]. Therefore, ED is regarded as a common mechanism for various CV disorders, and numerous clinical studies have shown that endothelial dysfunction can be an independent predictor of future cardiovascular disease (CVD) progression and acute thrombotic events [8–11].

Patients with autoimmune rheumatic diseases even in the absence of CV risk factors have an almost twofold increase in CV morbidity and mortality than the general population. It is thought that persistent systemic inflammation enhances CV risk through direct or indirect mechanisms leading to accentuation of existing risk pathways [12]. Such evidence has now been implemented in European guidelines (ESC 2016 and 2019, EULAR 2010 with 2015/2016 update) and risk scores [13–16]. Increased production of proinflammatory mediators and cytokines results in enhanced oxidative stress, the hallmark of both autoimmune diseases and atherosclerosis [17–20]. Elevated ROS generation, *via* activation of the transcription factor, nuclear factor *kappa*-light-chain-enhancer of activated B cell (NF-*κ*B) pathway, induces expression of inflammatory and immune genes (cytokines, chemokines, adhesion molecules, acute phase proteins, regulators of apoptosis, and cell proliferation). These mediators on one hand promote change in endothelial phenotype, known as endothelial activation; on the other hand, they potentiate inflammation *via* further recruitment of adaptive and innate immune cells and ROS generation, leading to persistence of inflammation and disease progression [21, 22]. It is thought that the destructive loop of oxidative stress and inflammation leads to development of endothelial dysfunction, a fundamental feature of atherosclerosis [23].

**Figure 1:**
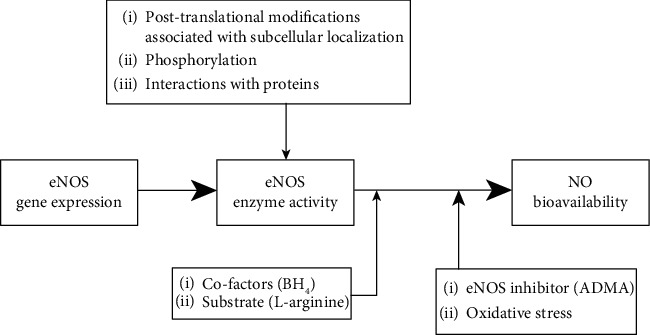
Balance between production and degradation of nitric oxide (NO) by oxidative stress determines endothelial NO bioavailability. Synthesis of NO can be regulated at the endothelial nitric oxide synthase (eNOS) gene expression level and eNOS enzymatic activity level. The eNOS activity depends also on substrate and cofactor availability and the presence of oxidative stress and endogenous inhibitor asymmetric dimethylarginine (ADMA). Adapted from Yang and Ming [177]. Abbreviations: eNOS: endothelial nitric oxide synthase; NO: nitric oxide; BH_4_: tetrahydrobiopterin; ADMA: assymetric dimethylarginine.

Due to the fact that atherosclerosis is a complex disease, no single mechanism can fully explain the endothelial dysfunction. However, decreased nitric oxide (NO) bioavailability with subsequent inability of endothelium to initiate vasodilatation and exhibit multiple antiatherogenic functions appears to play a major role [24]. Decreased NO bioavailability may result from its limited production and/or increased NO degradation by reactive oxygen species (ROS) (Figure 1). Reduced NO generation can be due to decreased endothelial NO synthase (eNOS) expression and/or activity, eNOS uncoupling, impaired NO-mediated signaling events, and oxidative stress. Among these mechanisms, the eNOS uncoupling has recently attracted the gaining attentions. However, there is scarcely no data in the literature on the role of the eNOS uncoupling in atherogenesis in autoimmune rheumatic diseases. The current review paper addresses this gap in literature.

## 2. Molecular Mechanisms of the eNOS Uncoupling: Pathophysiological Considerations and Potential Therapeutic Implications

### 2.1. eNOS Uncoupling (Figure 2): General Information

A number of studies have revealed that under pathological conditions, due to the enhanced oxidative stress, the eNOS may become dysfunctional resulting in production of superoxide instead of NO. Moreover, the expression of eNOS is increased by ROS through posttranscriptional and posttranslational modifications, although the NO bioavailability is reduced. This phenomenon contributes significantly to endothelial dysfunction and cardiovascular disease not only by reducing the NO generation but also by triggering the preexisting oxidative stress [25, 26].

Produced by the uncoupled eNOS, superoxide scavenges NO leading to the peroxynitrite formation. Both ROS exert multiple proatherogenic effects, including effects on eNOS function. Peroxynitrite oxidizes tetrahydrobiopterin (BH_4_), the eNOS cofactor to the trihydrobiopterin (BH_3_) radical, resulting in the eNOS uncoupling, perpetual superoxide production, and subsequent peroxynitrite formation [27]. It also reduces endothelial transport of L-arginine, the exclusive substrate for eNOS, and increases the rate of L-arginine efflux [28]. Peroxynitrite directly oxidizes the reduced glutathione (GSH), its endogenous scavenger, which plays a major role in the cellular defense against reactive oxygen species. Similarly, *via* nitration of superoxide dismutase (SOD), peroxynitrite inactivates the enzyme, leading to diminished antioxidant cellular defense mechanisms and increase in superoxide levels [29, 30]. Elevated superoxide levels are also the result of peroxynitrite action-induced protein phosphatase 2A (PP2A) activation, which leads in turn to the dephosphorylation of eNOS and therefore decrease in enzyme activity and subsequent NO generation [31, 32]. Peroxynitrite and superoxide, the known contributors to endothelial dysfunction, have also multiple indirect effects on the eNOS function. Peroxynitrite inactivates prostacyclin synthase (PGIS), an enzyme that catalyzes the isomerization of prostaglandin H_2_ to prostacyclin, widely known for its vasoprotective activity, therefore resulting in formation of vasoconstricting prostaglandins including thromboxane A_2_. Recent studies have shown that stimulation of thromboxane receptor (TPr) by thromboxane A_2_ and prostaglandin H_2_ promotes ROS formation in vascular smooth muscle cells and endothelial cells by activating nicotinamide adenine dinucleotide phosphate (NADPH) oxidase facilitating eNOS deactivation through increased oxidative stress [33, 34]. Both superoxide and peroxynitrite also oxidize low-density lipoproteins (LDL) forming oxidized LDL (ox-LDL), which in turn through the scavenger receptor, lectin-like oxidized low-density lipoprotein receptor-1 (LOX-1), downregulates the enzyme expression. Furthermore, ox-LDL stimulate NADPH oxidase and xanthine oxidase to produce ROS in excess, promoting a vicious cycle mechanism of oxidative stress and vascular damage [35, 36].

Numerous mechanisms have been proposed to play a role in the eNOS uncoupling in atherosclerosis: depletion of eNOS cofactor BH_4_, L-arginine deficiency, and increase in endogenous eNOS inhibitor, asymmetric dimethylarginine (ADMA) [37]. All these mechanisms are discussed below.

### 2.2. Asymmetric Dimethylarginine (ADMA)

ADMA is a naturally occurring amino acid formed from the proteolysis of methylated arginine residues in intracellular proteins that are posttranslationally modified by a class of enzymes known as protein-arginine methyl transferases (PRMTs). Following proteolysis, free methylarginines are released and subsequently converted to citrulline and dimethylamine by dimethylarginine dimethylaminohydrolase (DDAH). ADMA is a key NOS inhibitor—it competes with L-arginine for the binding site in the active center of NOS isoforms, thereby resulting in decreased NO generation [38–40]. Increased ADMA reduces NO bioavailability leading to subsequent inflammation and oxidative stress, the typical features of endothelial dysfunction, contributing substantially to cardiovascular risk [41, 42]. Therefore, increased ADMA levels have been associated with several risk factors and cardiovascular morbidity and mortality. Indeed, numerous studies have confirmed its role as an established independent predictor for cardiovascular events and all-cause cardiovascular mortality [43–45]. In animal models, ADMA levels correlated with vascular function and the degree of atherosclerosis, in humans with cholesterol levels [46, 47]. Elevated ADMA levels are largely due to increased PRMT activity or decreased DDAH activity. The regulation of gene expression and activity of PRMT and DDAH remains predominantly unclear. However, activities of both enzymes are redox sensitive. Oxidative stress has been shown to increase the activity of PRMTs and inhibit that of DDAH, resulting in elevated ADMA levels, which in turn via inhibition of NO synthesis and eNOS uncoupling enhance production of ROS [48]. Therefore, ADMA promotes superoxide production by eNOS, and the resulting oxidative stress upregulates ADMA levels [49]. Tumor necrosis factor-alpha (TNF-*α*) and high levels of glucose and homocysteine diminish DDAH activity via induction of oxidative stress. Similarly does oxidized low-density lipoprotein (ox-LDL) [50–52]. Increased ADMA in turn upregulates the LOX-1 expression, the main receptor for ox-LDL in endothelial cells, resulting in enhanced production of oxidized LDL and intracellular generation of reactive oxygen species, creating a vicious cycle mechanism [53, 54].

Negative regulation of NO synthesis can also be mediated through overproduction of methylated arginine analogues such as ADMA. Recently, many clinical studies have demonstrated that plasma levels of ADMA were elevated in RA patients regardless of the presence of cardiovascular disease [55–67]. Furthermore, ADMA has been reported to be related to indices of endothelial dysfunction or subclinical atherosclerosis in some [56, 65, 68], but not all, conducted studies [58, 60, 61, 67, 68]. In patients with high disease activity and no overt atherosclerotic disease or classic risk factors, high plasma ADMA levels significantly correlated with IMT [67], coronary flow reserve (CFR) [56], and pulse wave velocity (PWV) [58], whereas in those with evident atherosclerosis and CV risk factors, negative correlation between ADMA and FMD [65] and carotid IMT (cIMT) [62, 68] was found. In the latter subgroup of patients also, a positive relation was observed between the ADMA : SDMA ratio (suggested as the index of dimethylarginine dimethylaminohydrolase activity) and microvascular endothelial function [68] and arterial stiffness [62]. However, these relationships were not observed in RA patients with low and moderate disease activities—although structural and functional changes in vessels and heart were detected by means of multiple noninvasive, validated methods including cIMT, FMD, CFR, PWV, laser Doppler, and subendocardial viability ratio (SEVR), no associations between dimethylarginines and assessments of vascular morphology and function were found [56, 61, 65]. As an explanation of these findings, it has been suggested that inflammatory mechanisms responsible for synovial lesions might also occur in the vascular wall and promote the development of advanced atherosclerosis. Oxidative stress induced by proinflammatory cytokines has been shown to increase the activity of PRMTs and inhibit that of DDAH, resulting in elevated ADMA levels. The latter can be also due to increased endothelial cell turnover with potential liberation of ADMA during cell catabolism. Increased ADMA in turn contributes directly to oxidative stress by causing endothelial NOS uncoupling and switching it to a superoxide synthase. ADMA also significantly increases TNF-*α* levels in human endothelial cells and thus participates in the pathogenesis of vascular injury in RA [56, 62, 66, 67, 69]. Hence, these data indicate close interactions between endothelial injury and systemic inflammation.

### 2.3. Tetrahydrobiopterin (BH_4_)

BH_4_ is a critical cofactor for all the NOS isoforms and a regulator of their function [37]. It has been shown that NO generation and eNOS correlate closely with the intracellular concentration of BH_4_ [70]. It is synthesized de novo from guanosine triphosphate (GTP) in a multistep pathway that involves GTP cyclohydrolase I (GTPCH I), 6-pyruvoyltetrahydropterin synthase, and sepiapterin reductase, respectively. GTPCH I is a rate-limiting enzyme for BH_4_ biosynthesis and therefore plays a major role in controlling the NOS function [71, 72]. Many lines of evidence indicate that oxidative degeneration of BH_4_ by ROS leads to the eNOS uncoupling, reduction in NO bioavailability, and increased reactive oxygen species production [73, 74]. Indeed, oxidation of BH_4_ forms dihydrobiopterin (BH_2_) and biopterin. BH_2_ binds with fairly high affinity to eNOS without supporting its catalytic activity [75]. The uncoupled enzyme generates superoxide rather than NO leading to further limitation of BH_4_ availability. However, BH_2_ can be recycled to BH_4_ by dihydrofolate reductase (DHFR), which regulates the rate of BH_4_ regeneration [76]. Therefore, BH_4_ bioavailability is determined by enzymatic *de novo* synthesis, recycling, and oxidative degradation. There is little information on regulatory mechanisms of GTPCH and DHFR gene expression or activity. BH4 and high concentrations of BH_2_ inhibit GTPCH-1 and subsequently de novo synthesis of BH_4_, while insulin and mediators such as interferon gamma (IFN-*γ*), TNF-*α*, and interleukin-1 beta (IL-1*β*) can upregulate its activity and expression [77–80]. Expression of DHFR can be downregulated by angiotensin II [81]. It is thought that among these two enzymes, DHFR is critical to eNOS function, especially in cells that do not contain the apparatus required for efficient synthesis of BH_4_ or under conditions of low total biopterin levels, as recycling it can reduce eNOS-dependent oxidation of BH_4_ that would further decrease BH_4_ levels and enhance eNOS uncoupling [82]. In endothelial dysfunction and many models of cardiovascular disease, the BH_4_ levels have been found decreased. Therefore, recent studies have shown that pharmacological supplementation of BH_4_ improves vascular function in patients with diabetes, essential hypertension, and hypercholesterolemia and in chronic smokers [83–95].

Recently, it has been demonstrated that deficiency of BH_4_ may contribute in part to formation of the uncoupled eNOS. Indeed, a decrease in serum levels of BH_4_ in AIA rats compared to the control group was reported, and administration of BH_4_ restored endothelial function. Therefore, the authors suggested that eNOS contributes to amplification of oxidative stress in vasculature, and this contribution is mediated by the loss of BH_4_ availability [96, 97]. Beneficial effects of oral BH_4_ supplementation were then investigated in humans. Therapy with BH_4_ in patients with active RA improved endothelial function as assessed by vasodilatory response to reactive hyperemia. A decrease in BH_4_ levels in RA patients was attributed by the authors to increased expression and activation of inducible nitric oxide synthase (iNOS) in endothelial cells during chronic inflammation, which leads to eNOS uncoupling via limiting BH_4_ availability for eNOS. Also, ROS generated by myeloperoxidase released from activated neutrophils contribute to decreased BH_4_ levels via their oxidation to inactive BH_2_ [98, 99]. In an animal model of arthritis, serum BH_4_ levels besides supplementation can be increased upon administration of fluvastatin [97]. It has been reported in the general population that statins upregulate eNOS expression by stabilizing its mRNA and induce phosphorylation and activation of eNOS via the protein kinase Akt pathway. The authors showed that fluvastatin decreased expression of p22phox mRNA, a membrane-associated component of NADPH oxidase, resulting in inhibition of enzyme activity and decreased ROS generation. Therefore, they indicated that increase in BH_4_ availability due to decreased ROS production achieved with fluvastatin therapy prevents eNOS uncoupling [97, 100]. A similar mechanism of action presents etanercept, a TNF inhibitor [101].

Interestingly, methotrexate (MTX) inhibits NF-*κ*B activation through blockade of BH_4_ synthesis. It inhibits tetrahydrofolate reductase which recycles BH_2_ to BH_4_, leading to eNOS uncoupling and ROS production. In turn, increased ROS generation activates the Jun-N-terminal kinase (JNK) and JNK-dependent induction of tumor protein p53 (p53) and cyclin-dependent kinase inhibitor 1 (p21) resulting in decreased NF-*κ*B activation. Therefore, MTX may contribute to reduced BH_4_ bioavailability in the endothelium. However, the study investigating the impact of BH_4_ supplementation on endothelial function found no difference between patients on MTX and those not receiving MTX. Furthermore, patients treated with MTX had a greater increase in flow-mediated dilatation (FMD) following BH_4_ administration probably due to reduced levels of inflammation. Although the study was not powered to look at this difference, it has been reported recently that in activated T cells, inhibition of BH_4_ synthesis decreases production of the proinflammatory IFN-*γ* and increases production of the anti-inflammatory IL-4. MTX induces a similar shift and therefore downregulates expression of proinflammatory cytokines IL-1, IL-2, IL-6, and IFN-*γ* and upregulates expression of anti-inflammatory cytokines such as IL-4 and IL-10 in RA patients [98, 102, 103].

### 2.4. L-Arginine Deficiency

A semiessential amino acid L-arginine is the exclusive substrate for nitric oxide synthase, and its availability is one of the rate-limiting factors in cellular NO production [37]. Since reduction in L-arginine availability has emerged as an important mechanism underlying decreased NO bioavailability and endothelial dysfunction, many clinical and experimental studies during the past decade have shown beneficial effects of L-arginine supplementation in both animal studies and humans [104–112]. However, most recent studies are inconsistent with these findings showing no sustained effect or no effect of L-arginine administration on endothelial function [113–119]. This could be due to the complex biochemical metabolism of L-arginine [120]. L-Arginine is derived from dietary intake, protein breakdown, or endogenous de novo synthesis from L-citrulline catalyzed by the enzymes arginine-succinate synthase (ASS) and arginine-succinate lyase (ASL) [121]. Afterwards, it is converted into ornithine and urea by arginase, agmatine by L-arginine decarboxylase (ADC), and NO by NOS. Therefore, arginine metabolism and availability depend on the level of its dietary intake and endogenous synthesis on the one hand and the extent of catabolism on the other hand [122]. Whereas diminished bioavailability of NO is a common mechanism of various vascular disorders and endothelial dysfunction, the deficiency of L-arginine available for eNOS has been recently related to enhanced arginase activity [123]. The latter notion has been supported by findings in atherosclerosis and other cardiovascular disorders where arginase expression or activity has been found increased, suggesting that it plays a predominant role in these conditions [124–127]. However, information on exact regulatory mechanisms of arginase gene expression or activity is still missing. Its expression can be upregulated by proinflammatory factors: TNF-*α* and interferon-*γ*, ROS, oxidized LDL via the LOX-1 receptor, hyperglycaemia, thrombin, hypoxia, and angiotensin II. Arginase, both isoforms I and II, is expressed in endothelial and smooth muscle cells of the vascular wall and competes with NOS for the substrate L-arginine [128]. Increased activity of arginase leads to reduction in L-arginine availability for NOS, thereby decreasing the production of NO and resulting in eNOS uncoupling. Uncoupled enzyme produces superoxide instead of NO which further increases arginase activity and impair NO generation via oxidation of tetrahydrobiopterin [129–132]. Arginase also inhibits the L-arginine transport in endothelial cells further exacerbating L-arginine deficiency and downregulating NO production [133]. Moreover, arginase, by increased formation of polyamines and L-proline, stimulates vascular smooth muscle cell proliferation and extracellular matrix deposition, thereby contributing to intimal hyperplasia and remodeling processes. Similarly, promoting abnormal remodeling and neointimal hyperplasia reduced NO bioavailability [134, 135].

eNOS uncoupling resulting in reduced NO bioavailability and increased oxidative stress causes and aggravates dysregulation of endothelial function. New therapeutic strategies for atherosclerosis are aimed at preventing or reversing the endothelial dysfunction, before clinical manifestations and disease progression will occur. For better understanding of pathophysiology of endothelial dysfunction, novel pharmacological approaches focused on eNOS recoupling are being investigated. Several drugs currently in clinical use, inhibitors of the renin-angiotensin-aldosterone system, statins, and nebivolol, show many pleiotropic actions. Recently, it has been demonstrated that they may prevent or reverse the eNOS uncoupling and improve endothelial function and NO bioavailability in animal models. Similarly do resveratrol, sepiapterin, folic acid, AVE3085, and AVE9488 (enhancers of endothelial nitric oxide synthase acting on the eNOS gene transcription). All these compounds target eNOS through multiple direct and indirect mechanisms; however, the detailed mechanisms of their action are beyond the scope of this review and are comprehensively reviewed elsewhere [25, 37, 136]. Despite beneficial effects in animal models, applying these experimental results to clinical treatment still requires further studies and more extensive investigation.

Emerging evidence has suggested the deficiency of L-arginine available for eNOS as an etiology for endothelial dysfunction and has related it to enhanced arginase activity [137]. In an animal model of arthritis (adjuvant-induced arthritis), it has been shown that arginase II isoform expression and activity were significantly increased and correlated with disease activity [138, 139]. Nevertheless, high plasma arginase levels failed to correlate with plasma levels of IL-6. Although it is clearly recognized that systemic inflammation with increased proinflammatory cytokine production induces arginase expression, the exact regulatory mechanisms of enzyme activity or gene expression in the endothelial cells still remain elusive. In contrast, eNOS activity was found decreased with no change in its expression, and the authors attributed this discrepancy between eNOS activity and expression to decreased availability of the substrate for the enzyme. Moreover, they indicated that limiting L-arginine accessibility for NOS arginase upregulation contributes to enzyme uncoupling and endothelial dysfunction. Indeed, impaired endothelial function assessed by the vasodilating response to acetylcholine (Ach) was found in AIA rats, and arginase inhibition with a selective inhibitor N_w_-hydroxy-nor-L-arginine (nor-NOHA) restored vascular function. The effect of a curative treatment with nor-NOHA on vascular function of AIA rats was further investigated, and authors indicated that it is mediated by an increase in NOS activity and endothelium-derived hyperpolarizing factor (EDHF) production; a decrease in cyclooxygenase 2 (COX-2), thromboxane (TX), and prostaglandin I2 (PGI_2_) synthase and NADPH oxidase activities; and a decrease in superoxide production and secreted vascular endothelial growth factor (VEGF) levels. Both enhanced NOS activity and reduced superoxide production can be due to the decrease in vascular eNOS uncoupling, thanks to the beneficial effect of arginase inhibition and restored L-arginine bioavailability. Interestingly, arginase inhibition had no impact on disease severity assessed by clinical, histological, and radiological parameters, whereas it fully reversed endothelial dysfunction in AIA rats. Furthermore, plasma IL-6 levels did not correlate with endothelial dysfunction. Taking these findings into account, the authors conclude that endothelial dysfunction is not the consequence of the disease, at least in the chronic phase of the AIA model. Since the reduction of endothelial dysfunction seems to be possibly independent of RA disease activity, they indicated that the benefits provided by nor-NOHA are related to the direct modulation of endothelium-derived vasorelaxant pathways rather than an anti-inflammatory effect [138, 139]. It is noteworthy that this beneficial effect of arginase activity inhibition can also be obtained with statins, diclofenac, and etanercept [101, 140, 141].

## 3. Clinical Implications: eNOS Uncoupling and Autoimmune Rheumatic Disease

It is well established that patients with rheumatic autoimmune diseases are characterized by significantly increased prevalence of cardiovascular morbidity and mortality than the general population. The current European guidelines on cardiovascular disease (CVD) prevention in the clinical practice recommend to use a 1,5-factor multiplier for CV risk in rheumatoid arthritis as well as in other autoimmune inflammatory diseases. However, mechanisms of accelerated atherosclerosis in these diseases, especially in the absence of traditional risk factors, still remain unclear.

### 3.1. Systemic Lupus Erythematosus (SLE)

It is now clearly recognized that SLE patients are at high risk of developing CVD, and this excessive risk is especially pronounced in premenopausal women. Traditional risk factors do not fully account for this association, and the disease itself is considered an independent CV risk factor [142]. Therefore, precocious atherosclerosis is likely attributable to the consequences of inflammation, which is in line with observations that disease duration, higher damage index score, and less aggressive immunosuppression are associated with increased CVD burden in SLE patients. Till now, there are no biomarkers that predict CV events in SLE patients, and the known ones used to assess CV risk in the general population have limited or no value in SLE [143–145]. However, patients with SLE show enhanced endothelial dysfunction, which is regarded as a common mechanism for various CV disorders and considered the first step in atherosclerosis. Underlying mechanisms and its pathogenesis in SLE are still poorly understood [146, 147]. It is thought that the common denominator for multiple mechanisms contributing to the development of endothelial dysfunction is diminished activity of endothelial nitric oxide synthase and loss of nitric oxide production. Recent analyses have shown that SLE-specific circulatory factors, TNF-*α*, interleukin-17, interferons, ligand of cluster of differentiation 40 (CD40L), and C-reactive protein (CRP), lead to endothelial dysfunction via promotion of abnormal eNOS function and enhanced oxidative stress [148]. Among these mediators, type I interferons gained considerable attention. Continuous inflammatory production of interferon-alpha (IFN-*α*) and subsequent increased expression of IFN-*α*-regulated genes, referred as IFN signature, due to activation of plasmacytoid dendritic cells by immune complexes, consisting of autoantibodies in combination with deoxyribonucleic acid- (DNA-) or ribonucleic acid- (RNA-) containing autoantigens, have been reported in SLE patients. Studies on animals and humans have provided evidence that IFN accelerate atherosclerosis on multiple stages [149–151]. To date, no studies were conducted to determine direct effects of IFN on eNOS function and NO generation. However, IFN type I has been reported to have impact on enzyme cofactors, its specific transcription factors, and oxidative stress pathways [151]. Animal and human studies indicate that IFN-*α* leads to depletion of BH_4_ via oxidation, serving as a potential mediator of eNOS uncoupling and oxidative stress [152, 153]. There are also scarce studies investigating the role of interferon on L-arginine availability. However, a study conducted in patients with high-risk melanoma showed that therapy with pegylated IFN-*α* results in a marked decrease in the synthesis of NO and arginine availability [154, 155]. Information on impact on arginase activity is also missing. However, recently, a significant increase in serum arginase 1 activity was detected in the SLE patients. Its levels were positively correlated with the disease severity and IL-17. Furthermore, arginase 1 was found to enhance T helper 17 (Th17) cell differentiation both in vitro and in vivo, augmenting inflammation [156]. Although direct effect of these observations on eNOS function warrants further research, it is thought that inflammatory and immune process characteristics for SLE contribute to the development of premature atherosclerosis [157]. As reported, disease marker anti-Smith (anti-Sm) and anti-ribonucleoprotein (anti-RNP) antibodies stimulate IFN type I production by plasmacytoid dendritic cells [158]. Surprisingly, an inverse correlation between the presence of atherosclerosis in SLE (evaluated as arterial stiffness and presence of carotid plaque) and anti-nuclear antibodies was observed. Actually, patients with plaque had less frequent anti-Sm and/or anti-RNP antibodies than those without plaque [159]. Therefore, further studies are needed to clarify why patients with anti-nuclear antibodies have less pronounced subclinical atherosclerosis, even having more systemic and severe course of disease, interspersed with episodes of acute disease flares.

Besides IFN, anti-DNA autoantibodies are the hallmark of SLE. It has been implicated that anti-double-stranded DNA (anti-dsDNA) antibodies may have a role in the development of cardiovascular disease in SLE by enhancing ADMA production and by potentiating the inflammatory reaction. Indeed, it has been demonstrated *in vitro* that in the presence of anti-dsDNA, methylation of arginine residues in proteins by PRMT I is increased; therefore, anti-dsDNA antibodies may be a trigger for enhanced ADMA production in SLE [160]. Results of in vitro studies were confirmed by findings observed in vivo, where there are high plasma levels of complement (C3 and C4), measures of disease activity and organ damage, CV events, and prednisone use [161, 162]. Although ADMA is significantly associated with risk factors for CVD in the general population, no such correlation was found in SLE patients [161, 162].

Subclinical atherosclerosis in SLE has been reported and described by different methods. In SLE patients without CVD, the ADMA was independently associated with the coronary calcium score and arterial stiffness [159, 162]. Nevertheless, no association with the presence or extent of carotid atherosclerosis (assessed by carotid ultrasonography—intima-media thickness (IMT) and plaque) was found [159, 162]. These inconsistent findings are attributed by authors to differential effect of ADMA on distinct vascular beds. There are two major mechanisms proposed underlying vascular disease in SLE: IFN-induced reduction of endothelial cell proliferation and survival with subsequent impaired repair and remodeling and ADMA-induced inhibition of eNOS [159]. Nevertheless, further studies are needed to investigate the role of the NO pathway and its components in atherogenesis in SLE.

### 3.2. Rheumatoid Arthritis (RA)

Similar to SLE, endothelial dysfunction has been reported in RA patients, even in the very early stages of disease [163]. Since it has been speculated that RA-related inflammation might contribute to endothelial dysfunction, anti-TNF therapy has been shown to improve vascular function, which strongly indicates involvement of systemic inflammation in the development of premature atherosclerosis [164]. Recent studies on animals showed that endothelial function in adjuvant-induced arthritis (AIA) rats is significantly depressed without any histologic damage, supporting the idea that endothelial dysfunction occur before overt vascular damage [96]. The mechanism of endothelial dysfunction in RA remains still incompletely understood, but decreased NO bioavailability along with increased ROS production has been suggested. Besides NADPH oxidase, uncoupling eNOS has been identified as an important source of ROS and its expression was significantly increased at both messenger RNA (mRNA) and protein levels in AIA rats. Furthermore, incubation of homogenates of AIA rat aortas with L-arginine led to overproduction of superoxide.

Although RA disease-related inflammation may contribute to elevated ADMA levels and increased CVD risk in RA, the association between ADMA and disease activity has been an issue of debate, as previous studies are heterogenous in results. It has been demonstrated that baseline disease status (mainly elevated erythrocyte sedimentation rate (ESR) and also CRP) and cumulative inflammatory burden (6 years of follow-up) had a positive correlation with current ADMA levels as—according to authors—patients with longer periods of uncontrolled disease are more prone to develop endothelial dysfunction due to the higher cumulative inflammatory burden on the vasculature [165]. A few studies have reported a positive correlation between ADMA and inflammatory markers (CRP and ESR), disease activity (DAS 28) and duration, and clinical parameters of disease status (tender and swollen joints, morning rigid) independently of the presence of classical risk factors and CVD [61, 64–66, 165–168], not confirmed by other studies [60, 61, 63, 66, 169]. Similar results were obtained concerning RA disease-specific markers—rheumatoid factor (RF) and anti-citrullinated protein antibodies (ACPA) [60, 61, 63–66, 168]. Interestingly, no association was found with traditional risk factors [55, 68], apart from homeostatic model assessment (HOMA) referred to as the indicator of insulin resistance, being the only independent predictor of elevated ADMA levels in RA patients [169]. Additionally, ADMA has been shown to correlate with other biomarkers of vascular dysfunction such as endothelial progenitor cell (EPC) count [67].

The studies investigating a possible impact of the disease-modifying antirheumatic drugs (DMARDs) on ADMA levels also provided conflicting results. In a small prospective study conducted on treatment-naïve patients with early RA, a significant decrease in ADMA serum levels after 12 months of immunosuppressive treatment with synthetic and biologic DMARDs along with glucocorticoids was reported [60]. However, no increase in carotid IMT was observed after 12 months of DMARD therapy. These findings were not confirmed by another study performed in a similar subgroup of patients treated for 18 months with either methotrexate or adalimumab [170]. Although an improvement in CFR was found, both carotid IMT and plasma ADMA levels did not show significant changes after therapy. The lack of effect of methotrexate and TNF inhibitors (etanercept, adalimumab, and infliximab) on plasma concentrations of ADMA was also demonstrated in long-standing RA patients [58, 61]. Data from studies determining the impact of short-term anti-TNF administration are also inconsistent. One report described similar to baseline ADMA values after 2 weeks and 3 months of anti-TNF treatment with etanercept, infliximab, or adalimumab [167], whereas others demonstrated a significant reduction of dimethylarginine in the group of patients receiving etanercept or adalimumab [166, 171]. In the latter study by Spinelli et al., besides a decrease in ADMA plasma concentrations, anti-TNF therapy restored circulating endothelial progenitor cell levels, although a not significant increase of FMD was observed. Finally, it has been shown that TNF inhibitors improved the L-arginine/ADMA ratio due to the increase in L-arginine, and the L-arginine/ADMA ratio was longitudinally related to PWV after initiation of anti-TNF-*α* therapy [58]. Acute and chronic oral treatments with glucocorticoids have also different effects on arginine metabolites; while acute prednisolone therapy has no impact, chronic prednisolone treatment reduces ADMA and SDMA plasma concentrations [57].

Given the evident role of TNF in atherosclerosis and RA pathogenesis and its inhibitory effect on DDAH leading to ADMA accumulation, a beneficial effect of TNF inhibition has been postulated; however, results of conducted studies did not demonstrate a consistent decrease in ADMA levels with subsequent improvement in vascular morphology and function suggesting that the ADMA level does not seem to be a straightforward indicator of endothelial dysfunction and subclinical atherosclerosis in rheumatic diseases. Due to the complexity of the processes observed in RA and atherosclerosis, contributions of traditional and disease-related risk factors cannot be excluded as well as other mechanisms of DMARD action compared with increased NOS activity/expression. The heterogeneity of the study population and methods used to assess subclinical atherosclerosis may also account for the lack of concordance of the results and limit the usefulness of ADMA as a marker for atherosclerotic risk stratification [61, 165, 171].

In accordance with results from animal studies, an increase in plasma arginase activity with a significant decrease in arginine bioavailability was reported in patients with RA [55]. Similar observations were made regarding the catabolic product of arginase (L-ornithine) and catabolic product of NOS (L-citrulline). Interestingly, it has been shown that elevated arginase activity was associated with prior history of CVD in a subgroup of patients with RA, but it did not show any correlation with traditional risk factors. In contrast to animal studies, significant increase in arginase activity seemed to be independent of disease activity; however, patients in the analyzed cohort had relatively low RA disease activity reflected by Disease Activity Score (DAS28). On the other hand, systemic inflammatory conditions can increase arginase expression in endothelial and immune cells, and therefore, authors indicate that elevated arginase levels can be due to higher turnover of these cells. Although the relationship between systemic inflammation in RA and arginase activity warrants further research, authors avail this disconnection between arginase activity and RA disease activity for the clinical practice and proposed arginase activity as a potential biomarker of increased CVD risk independent of the patient's disease state [55].

### 3.3. Primary Sjogren Syndrome (pSS)

There is mounting evidence that primary Sjogren syndrome, similar to SLE and RA, has increased morbidity of CVD [172]. However, limited evidence is available for primary SS regarding premature atherosclerosis and endothelial dysfunction. Previous studies examining subclinical CVD measured by different techniques were heterogenous in results. Nevertheless, their results indicate a subclinical vascular damage that would explain higher CV risk [173]. There are scarcely no studies determining the eNOS function and NO generation in pSS. However, some recent data shows increased oxidative stress in pSS and association of disease with IFN-I signature, which could exert indirect effects as described above [174–176]. Therefore, pSS emerges due to the similarity to SLE and RA and also due to the fact that most patients are out on medication, as an interesting model to study atherosclerosis in autoimmune diseases.

## 4. Conclusions

The role of oxidative stress has been well established in the development and progression of atherosclerosis, and eNOS uncoupling appears to be an important mechanism contributing to increased ROS generation. Moreover, eNOS uncoupling is also mediated by excessive ROS formation (“ROS-induced ROS formation”).

Chronic systemic inflammation is considered an independent CV risk factor, and it contributes significantly to oxidative stress. Due to the close interaction between inflammation and oxidative stress, autoimmune rheumatic diseases are associated with increased CV morbidity and mortality even when traditional risk factors are absent. However, the exact role of eNOS uncoupling in premature atherogenesis in rheumatic diseases is still not fully elucidated. Similarly, there is scarcity of data on the interactions between the NO metabolic pathway and disease-related factors. The best-studied mechanisms thus far are the depletion of eNOS cofactor BH_4_, L-arginine deficiency, and increase in endogenous eNOS inhibitor, ADMA. Therefore, the thorough understanding of molecular mechanisms underlying impaired NO bioavailability and eNOS dysfunction may help to identify the best and most effective approach to prevent and manage CV complications in rheumatic diseases.

## Figures and Tables

**Figure 2:**
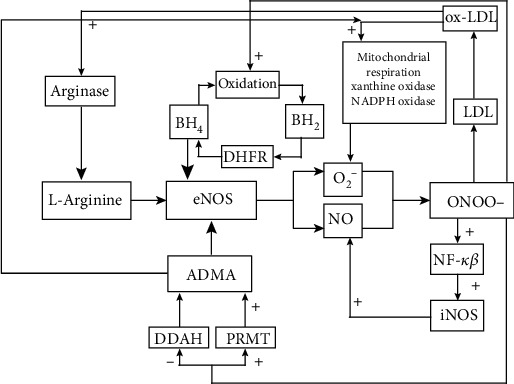
Mechanisms of endothelial nitric oxide synthase (eNOS) uncoupling in endothelial dysfunction. A depletion of eNOS cofactor tetrahydrobiopterin (BH_4_), an L-arginine deficiency, and an increase in endogenous eNOS inhibitor, asymmetric dimethylarginine (ADMA), leads to eNOS uncoupling. Produced by the uncoupled enzyme, superoxide scavenges nitric oxide (NO) leading to the peroxynitrite formation. Peroxynitrite (1) oxidizes BH_4_, resulting in the eNOS uncoupling and perpetual superoxide production and subsequent peroxynitrite formation; (2) oxidizes low-density lipoproteins (LDL) forming oxidized LDL (ox-LDL) which in turn through the scavenger receptor, lectin-like oxidized low-density lipoprotein receptor-1 (LOX-1), downregulate the enzyme expression. Furthermore, ox-LDL stimulate nicotinamide adenine dinucleotide phosphate oxidase (NADPH oxidase) and xanthine oxidase to produce reactive oxygen species (ROS) in excess and increase arginase activity leading to reduction in L-arginine availability for nitric oxide synthase (NOS) and subsequent eNOS uncoupling and impaired NO generation. Moreover, arginase via increased formation of polyamines and L-proline stimulates vascular smooth muscle cell proliferation and extracellular matrix deposition, thereby contributing to intimal hyperplasia and remodeling processes; (3) nitrosylates the cationic amino acid transporter, therefore inhibiting the L-arginine transport in endothelial cells and increasing the rate of arginine efflux; (4) increases the activity of protein-arginine methyl transferase (PRMTs) and inhibits that of dimethylarginine dimethylaminohydrolase (DDAH), resulting in elevated ADMA levels, which in turn via inhibition of NO synthesis and eNOS uncoupling enhance production of ROS. Oxidative stress in turn upregulates ADMA levels; (5) through activation of transcription factor, nuclear factor kappa-light-chain-enhancer of activated B cells (NF-*κ*B) induces expression of inducible nitric oxide synthase (iNOS), arginase, and inflammatory and immune genes (cytokines, chemokines, adhesion molecules, acute phase proteins, regulators of apoptosis, and cell proliferation) potentiating inflammation via further recruitment of adaptive and innate immune cells and ROS generation, leading to persistence of inflammation and disease progression. Abbreviations: eNOS: endothelial nitric oxide synthase; NO: nitric oxide; BH_4_: tetrahydrobiopterin; ADMA: asymmetric dimethylarginine; O_2_^−^: superoxide; ONOO-: peroxynitrite; BH_2_: dihydrobiopterin; DHFR: dihydrofolate reductase; ox-LDL: oxidized LDL; NADPH oxidase: nicotinamide adenine dinucleotide phosphate oxidase; LDL: low-density lipoproteins; iNOS: inducible nitric oxide synthase; NF-*κ*B: nuclear factor kappa light-chain-enhancer of activated B cells; DDAH: dimethylarginine dimethylaminohydrolase; PRMT: protein arginine methyl transferase.
